# Six New Coumarin Glycosides from the Aerial Parts of *Gendarussa vulgaris*

**DOI:** 10.3390/molecules24081456

**Published:** 2019-04-12

**Authors:** Yanjun Sun, Meiling Gao, Haojie Chen, Ruijie Han, Hui Chen, Kun Du, Yanli Zhang, Meng Li, Yingying Si, Weisheng Feng

**Affiliations:** 1 Collaborative Innovation Center for Respiratory Disease Diagnosis and Treatment & Chinese Medicine Development of Henan Province, Henan University of Chinese Medicine, Zhengzhou 450046, Henan, China; gaoxiaomei6266@126.com (M.G.); CHj3928@126.com (H.C.); 18638221936@126.com (R.H.); chenhuiyxy@hactcm.edu.cn (H.C.); qqninenine@hotmail.com (K.D.); zyl2013hnzy@163.com (Y.Z.); limeng31716@163.com (M.L.); yingying8690@163.com (Y.S.); 2 School of Pharmacy, Henan University of Chinese Medicine, Zhengzhou 450046, Henan, China

**Keywords:** *Gendarussa vulgaris*, coumarin glycoside, cytotoxic activity

## Abstract

Six new coumarin glycosides, genglycoside A–F (**1**–**6**), were isolated from the aerial parts of *Gendarussa vulgaris*, along with ten known analogues (**7**–**16**). Their structures were unambiguously established on the basis of extensive spectroscopic data and HPLC analysis. The cytotoxic activities of all isolated compounds were evaluated by MTT assay. Compound **12** showed the most potent cytotoxicity in Eca-109, MCF-7, and HepG2 cell lines. By the preliminary structure–activity relationships, it was firstly discovered that the glycosylation or esterification at 7,8-dihydroxy or 7-hydroxy drastically reduced the cytotoxic activity of the parent coumarin.

## 1. Introduction

**Figure 1:**
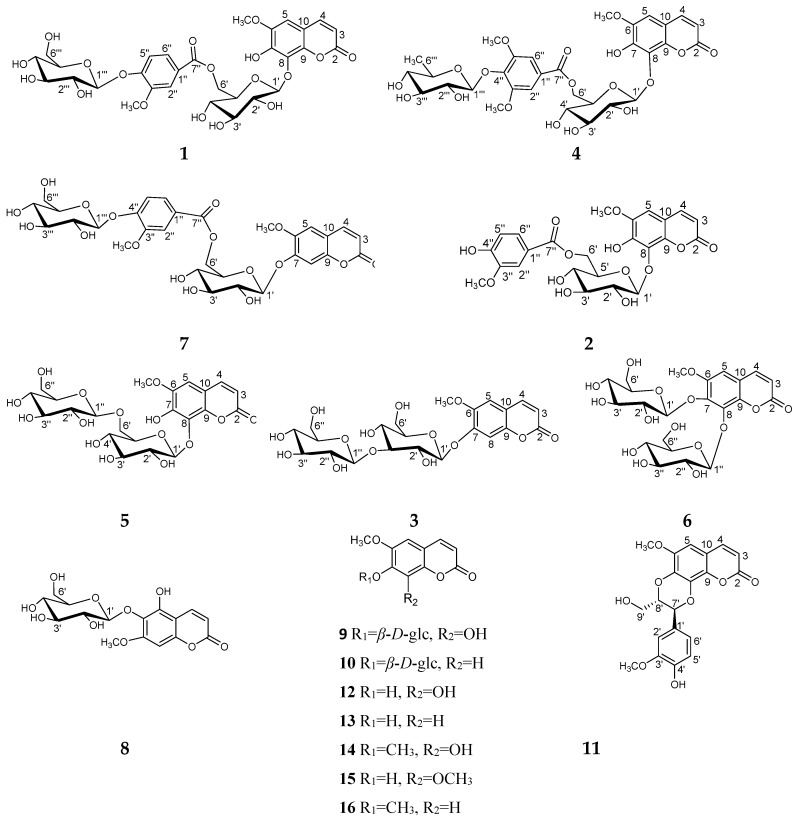
The chemical structures of compounds **1**–**16** from *Gendarussa vulgaris*.

*Gendarussa vulgaris* Nees, which belongs to the family of Acanthaceae, is an evergreen dwarf shrub mainly distributed in China, India, Sri Lanka, and the Malay Peninsula [1]. As an important medicinal plant, it has been described in *Chinese Pharmacopoeia 2015*, *Supplement to the Compendium of Materia Medica*, *Luchuan Materia Medica*, *Lingnan Medical Records*, etc. Its aerial parts (called Xiaobogu in Chinese) are frequently used for the treatment of fascia fracture, traumatic injury, rheumatism, and ostalgia, blood stasis menstrual block, and postpartum abdominal pain [2]. Previous chemical investigations on *G. vulgaris* revealed the presence of bioactive alkaloids, flavonoids, phenylpropanoids, steroids, and triterpenes [1,2]. Naturally occurring coumarins have exhibited a broad spectrum of pharmacological actions, including as an anticoagulant [3], CNS stimulant [4], antioxidant [5], antiviral [6], hepatoprotective [7], anti-inflammatory [8], anticancer [9], and cyclooxygenase, lipooxygenase, cholinesterase (ChE), and monoamine oxidase (MAO) inhibitory activities [9,10], antimutagenic [10], etc. As potential therapeutic drugs against cancer, coumarins have not only exhibited obvious anti-proliferative activity in malignant melanoma, prostate cancer, and renal cell carcinoma in some clinical trials [11], but also very rare cardiotoxicity, nephrotoxicity, dermal toxicity, and other MDR (multi-drug resistance) side effects [9]. In our search for cytotoxic natural products from the aerial parts of *G. vulgaris*, six new coumarin glucosides (**1**–**6**) were obtained together with ten known analogues (**7**–**16**). Details of the isolation, structure elucidation, and cytotoxicity of all isolated compounds against Eca-109, MCF-7, and HepG2 cell lines are described here (Figure 1).

## 2. Results and Discussion

The EtOH extract of the aerial parts of *G. vulgaris* was partitioned between PE, EtOAc, *n*-BuOH, and water, respectively. The EtOAc and *n*-BuOH layers were fractionated and purified by repeated column chromatography, allowing the isolation of sixteen coumarins (**1**–**16**), including six new coumarin glycosides, genglycoside A–F (**1**–**6**), along with ten known analogues. The known metabolites were identified as indidene F (**7**) [12], isofraxetin 6-*O*-*β*-d-glucopyranoside (**8**) [13], fraxin (**9**) [14], scopoletin 7-*O*-*β*-d-glucopyranoside (**10**) [15], cleomiscosin A (**11**) [16], fraxetin (**12**) [17], scopoletin (**13**) [18], fraxidin (**14**) [19], isofraxidin (**15**) [17], scoparone (**16**) [20], by comparison of their spectroscopic data with values reported in the literature.

**Figure 2:**
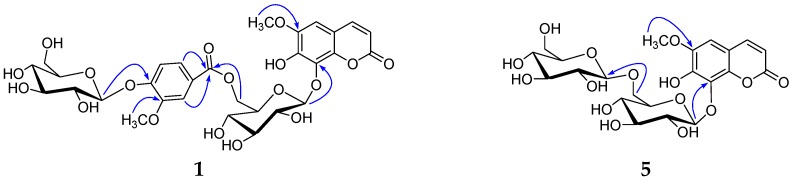

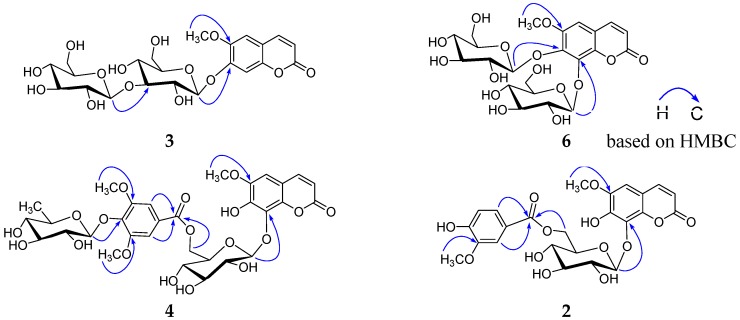
Key HMBC correlations of compounds **1**–**6**.

Compound **1** was obtained as a white, amorphous powder. The positive HR-ESI-MS spectrum revealed an [M + K]^+^ peak at *m*/*z* 721.1381 (calcd. 721.1382 for C_30_H_34_O_18_K), suggesting a molecular formula of C_30_H_34_O_18_ with fourteen degrees of unsaturation. Its IR spectrum showed the presence of hydroxyl (3384 cm^−1^), carbonyl (1712 cm^−1^), and aromatic ring (1610, 1509 cm^−1^). The UV spectrum showed the maximum absorptions at 207, 291, and 335 nm. The ^1^H NMR spectrum (Table 1, see Figure S1 in Supplementary Materials) showed two cis-olefinic protons *δ* 7.80 (1H, d, *J* = 9.5 Hz), 6.13 (1H, d, *J* = 9.5 Hz), and one aromatic proton *δ* 6.93 (1H, s), suggesting the existence of one 6,7,8-trisubstituted coumarin skeleton. One vanilloyl (4-hydroxy-3-methoxybenzoyl) group was deduced by one ABX system of aromatic protons *δ* 7.28 (1H, d, *J* = 1.9 Hz), 7.05 (1H, d, *J* = 8.5 Hz), 7.18 (1H, dd, *J* = 8.5, 1.9 Hz), and one aromatic methoxy group *δ* 3.74 (3H, s). Furthermore, the ^1^H NMR spectrum also displayed one remaining aromatic methoxy group *δ* 3.77 (3H, s), two sugar anomeric protons *δ* 5.06 (1H, d, *J* = 5.3 Hz), 5.12 (1H, d, *J* = 5.3 Hz). d-glucose was identified by acid hydrolysis and HPLC analysis. The *β*-configuration of d-glucose was determined by the large coupling constants (*J* = 5.3, 5.3 Hz) of the anomeric protons and the chemical shifts (*δ* 103.2, 99.6) of the anomeric carbons. The ^13^C-NMR spectrum (Table 2, see Figure S2 in Supplementary Materials) also revealed one coumarin skeleton including one carbonyl group *δ* 160.1, two olefinic carbons *δ* 111.9, 144.6, one benzene ring *δ* 110.0, 104.8, 145.3, 148.4, 131.0, 142.8, besides one methoxyl group *δ* 56.1, one vanilloyl group *δ* 122.8, 112.4, 148.4, 150.5, 114.0, 123.6, 165.0, 55.5, and two glucopyranosyl groups *δ* 103.2, 73.7, 76.2, 70.4, 74.2, 64.0, 99.6, 73.1, 76.8, 69.7, 77.3, 60.8. These spectroscopic data indicated that compound **1** was a coumarin glucoside derivative. The aglycone was identified as fraxetin, by comparison of its NMR data with those reported in the literature [17]. By the HMBC correlations (Figure 2) of the anomeric protons *δ* 5.06 (1H, d, *J* = 5.3 Hz, H-1′) and 5.12 (1H, d, *J* = 5.3 Hz, H-1′′′) with C-8 (*δ* 131.3) and C-4′′ (*δ* 150.5), two glucopyranosyl groups were linked to C-7 and C-4′′ respectively. The HMBC correlation of the oxymethylene proton *δ* 4.19 (1H, dd, *J* = 11.8, 7.5 Hz, H-6′) with C-7′′ (*δ* 165.0), indicated that the 6-OH of inner glucopyranosyl group was esterified by vanillic acid. The remaining methoxy group was located at C-6, based on the HMBC correlation between the methoxy group protons *δ* 3.77 (3H, s) and C-6 (*δ* 145.3). Thus, compound **1** was established as 8-[6-(4-*O*-*β*-d-glucopyranosyloxy-3-methoxybenzoyl)]-*O*-*β*-d-glucopyranosyloxy-6-methoxy-7-hydroxycoumarin, and named genglycoside A.

Compound **2** was obtained as a white, amorphous powder. Its IR spectrum showed the presence of hydroxyl (3359 cm^−1^), carbonyl (1710 cm^−1^), and aromatic ring (1602, 1505 cm^−1^). The UV spectrum showed the maximum absorptions at 205, 295, and 345 nm. Its ^1^H and ^13^C NMR (Table 1 and Table 2, see Figures S5 and S6 in Supplementary Materials) were quite similar to those of **1**, except that one glucopyranosyl group disappeared in **2**. This was further supported by its HR-ESI-MS, which gave an [M + Na]^+^ quasi-molecular ion peak *m/z* 543.1097 (calcd. for C_24_H_24_O_13_Na, 543.1115), being 162 mass-units less than that of **1**. The HMBC correlation (Figure 2) between the sugar anomeric proton *δ* 4.94 (1H, d, *J* = 7.7 Hz, H-1′) and C-8 (δ 131.5), indicated that the glucopyranosyl group was also attached to C-8. In addition, the HMBC correlation between the oxymethylene proton *δ* 4.14 (1H, dd, *J* = 11.8, 7.5 Hz, H-6′) and C-7′′ (*δ* 165.3), suggested that the 6-OH of glucopyranosyl group was esterified by vanillic acid. Thus, compound **2** was identified as 8-[6-(3-hydroxy-4-methoxybenzoyl)] -*β*-d-glucopyranosyloxy-6-methoxy-7-hydroxycoumarin, and named genglycoside B.

Compound **3** was obtained as a white, amorphous powder. The positive HR-ESI-MS spectrum revealed an [M + Na]^+^ peak at *m*/*z* 539.1396 (calcd. 539.1377 for C_22_H_28_O_14_Na), suggesting a molecular formula of C_22_H_28_O_14_ with nine degrees of unsaturation. Its IR spectrum showed the presence of hydroxyl (3333 cm^−1^), carbonyl (1700 cm^−1^), and aromatic ring (1612, 1512 cm^−1^). The UV spectrum showed the maximum absorptions at 204, 290, and 340 nm. The ^1^H NMR spectrum (Table 1, see Figure S9 in Supplementary Materials) indicated the presence of one 6,7-disubstituted coumarin skeleton *δ* 7.96 (1H, d, *J* = 9.6 Hz), 6.33 (1H, d, *J* = 9.6 Hz), 7.30 (1H, s), 7.17 (1H, s), anomeric protons of two glucopyranosyl groups *δ* 5.21 (1H, d, *J* = 7.4 Hz), 4.35 (1H, d, *J* = 7.8 Hz), one methoxy group *δ* 3.81 (3H, s). The ^13^C NMR spectrum (Table 2, see Figure S10 in Supplementary Materials) exhibited one coumarin skeleton including one carbonyl group *δ* 160.4, two olefinic carbons *δ* 113.4, 144.1, one benzene ring *δ* 112.4, 110.0, 146.0, 148.9, 103.1, 148.9, besides two sets of glucopyranosyl groups *δ* 99.0, 71.8, 87.4, 67.9, 76.5, 61.1, 103.9, 73.8, 75.9, 70.1, 76.9, 60.4, one methoxy group *δ* 56.1. d-glucose was also identified by the same analytical method as compound **1**. The large coupling constants (7.4, 7.8 Hz) of anomeric protons allowed the identification of two *β*-glucopyranosyl moieties. The HMBC cross peaks (Figure 2) of the anomeric proton *δ* 5.21 (1H, d, *J* = 7.4 Hz, H-1′) and 4.35 (1H, d, *J* = 7.8 Hz, H-1′′) with C-7 (*δ* 148.9) and C-3′ (*δ* 87.4), respectively, indicated that one glucopyranosyl group was linked to C-7 of the aglycone and the other was substituted at C-3′ of the inner glucopyranosyl group. The methoxy group was located at C-6, based on the HMBC correlation between methoxy group protons *δ* 3.81 (3H,s) and C-6 (*δ* 146.0). Thus, compound **3** was established as 7-[(3-*O-β*-d-glucopyranosyl-*β*-d-glucopyranosyl)oxy]-6-methoxycoumarin, and named genglycoside C.

Compound **4** was obtained as a white, amorphous powder. Its IR spectrum showed the presence of hydroxyl (3377 cm^−1^), carbonyl (1709 cm^−1^), and an aromatic ring (1603, 1501 cm^−1^). The UV spectrum showed the maximum absorptions at 208, 292, and 343 nm. Its ^1^H and ^13^C NMR (Table 1 and Table 2, see Figures S13 and S14 in Supplementary Materials) were quite similar to those of **1**, except for the appearance of one syringoyl group and one rhamnopyranosyl moiety in **4**, respectively, instead of one vanilloyl group and one glucopyranosyl moiety found in **1.** This was further supported by its HR-ESI-MS, which gave an [M + Na]^+^ quasi-molecular ion peak *m/z* 719.1794 (calcd. for C_31_H_36_O_18_Na, 719.1800), being 14 mass-units more than that of **1**. The syringoyl (4-hydroxy-3,5-dimethoxybenzoyl) group was deduced by two aromatic protons *δ* 7.03 (2H, s), two aromatic methoxy groups *δ* 3.79 (6H, s), combined with the HMBC correlation of the aromatic protons *δ* 7.03 (2H, s) with the carbonyl group *δ* 167.0. d-glucose and l-rhamnose were also identified by the same HPLC analysis as compound **1**. The large coupling constant (7.8 Hz) of anomeric proton allowed the identification of *β*-glucopyranosyl moiety. The α configuration for the l-rhamnopyranosyl unit was established by comparison of its NMR data with the literature values [21]. The rhamnopyranosyl group was confirmed by six aliphatic carbons *δ* 103.4, 72.3, 73.6, 72.2, 72.0, 18.0. By the HMBC correlations (Figure 2) of anomeric protons *δ* 5.11 (1H, d, *J* = 7.8 Hz, H-1′) and *δ* 5.38 (1H, br.s, H-1′′′) with C-8 (*δ* 132.2) and C-4′′ (*δ* 139.7), the glucopyranosyl and rhamnopyranosyl groups were linked to C-8 and C-4′′, respectively. The HMBC correlations of the methylene protons *δ* 4.60 (1H, m, H-6′), 4.41 (1H, m, H-6′) with C-7′′ (*δ* 167.0), indicated that the 6-OH of inner glucopyranosyl was esterified by syringic acid. The remaining methoxy group was located at C-6, based on the HMBC correlation between methoxy group protons *δ* 3.80 (3H, s) and C-6 (*δ* 147.2). Thus, compound **4** was established as 8-[6-(4-*O*-*α*-l-rhamnopyranosyloxy-3,5-dimethoxybenzoyl)]-*O*-*β*-d-glucopyranosyloxy-6-methoxy-7-hydroxycoumarin, and named genglycoside D.

Compound **5** was obtained as a white, amorphous powder. The positive HR-ESI-MS spectrum revealed an [M + Na]^+^ peak at *m*/*z* 555.1320 (calcd. 555.1326 for C_22_H_28_O_15_Na), suggesting a molecular formula of C_22_H_28_O_15_ with nine degrees of unsaturation. Its IR spectrum showed the presence of hydroxyl (3378 cm^−1^), carbonyl (1706 cm^−1^), and aromatic ring (1607, 1509 cm^−1^). The UV spectrum showed the maximum absorptions at 206, 292, and 339 nm. The ^1^H NMR spectrum (Table 1, see Figure S17 in Supplementary Materials) indicated the presence of one 6,7,8-trisubstituted coumarin skeleton *δ* 7.88 (1H, d, *J* = 9.5 Hz), 6.22 (1H, d, *J* = 9.5 Hz), 7.02 (1H, s), anomeric protons of two glucopyranosyl groups *δ* 4.97 (1H, d, *J* = 7.8 Hz), 4.06 (1H, d, *J* = 7.8 Hz), one methoxy group *δ* 3.81 (3H, s). The ^13^C NMR spectrum (Table 2, see Figure S18 in Supplementary Materials) also exhibited one coumarin skeleton including one carbonyl group *δ* 160.2, two olefinic carbons *δ* 111.2, 144.8, one benzene ring *δ* 110.1, 105.0, 145.3, 143.7, 131.3, 142.7, besides two sets of glucopyranosyl groups *δ* 103.6, 73.8, 76.4, 69.5, 76.5, 67.7, 103.0, 73.5, 76.2, 69.8, 76.6, 60.8, one methoxy group *δ* 56.1. d-glucose was also identified by the same analytical method as compound **1**. The large coupling constants (7.8, 7.8 Hz) of anomeric protons allowed the identification of two *β*-glucopyranosyl moieties. The HMBC cross peaks (Figure 2) of the anomeric protons *δ* 4.97 (1H, d, *J* = 7.8 Hz, H-1′), 4.06 (1H, d, *J* = 7.8 Hz, H-1′′) with C-8 (*δ* 131.3) and C-6′ (*δ* 67.7), indicated that one glucopyranosyl group was linked to C-8 of the aglycone and the other was substituted at C-6′ of the inner glucopyranosyl group. The methoxy group was located at C-6, based on the HMBC correlation between methoxy group *δ* 3.81 (3H,s) and C-6 (*δ* 145.3). Thus, compound **5** was established as 8-[6-(*β*-d-glucopyranosyloxy)]-*O*-*β*-d-glucopyranosyloxy-6-methoxy-7-hydroxycoumarin, and named genglycoside E.

Compound **6** was obtained as a white, amorphous powder. The positive HR-ESI-MS spectrum revealed an [M + Na]^+^ peak at *m*/*z* 555.1320 (calcd. 555.1326 for C_22_H_28_O_15_Na), suggesting a molecular formula of C_22_H_28_O_15_ with nine degrees of unsaturation. Its IR spectrum showed the presence of hydroxyl (3327 cm^−1^), carbonyl (1682 cm^−1^), and aromatic ring (1608, 1506 cm^−1^). The UV spectrum showed the maximum absorptions at 207, 292, and 338 nm. The ^1^H NMR spectrum (Table 1, see Figure S21 in Supplementary Materials) indicated the presence of one 6,7,8-trisubstituted coumarin skeleton *δ* 7.94 (1H, d, *J* = 9.5 Hz), 6.39 (1H, d, *J* = 9.5 Hz), 7.14 (1H, s), anomeric protons of two glucopyranosyl groups *δ* 5.26 (1H, d, *J* = 7.8 Hz), 5.18 (1H, d, *J* = 7.8 Hz), one methoxy group *δ* 3.81 (3H, s). The ^13^C NMR spectrum (Table 2, see Figure S22 in Supplementary Materials) exhibited one coumarin skeleton including one carbonyl group *δ* 159.9, two olefinic carbons *δ* 114.9, 144.2, one benzene ring *δ* 114.4, 105.9, 149.6, 141.3, 136.1, 142.4, besides two sets of glucopyranosyl groups *δ* 102.6, 74.0, 76.4, 69.9, 77.6, 60.7, 102.5, 74.0, 76.4, 69.9, 77.5, 60.7, one methoxy group *δ* 56.6. d-glucose was also identified by the same analytical method as compound **1**. The large coupling constants (7.8, 7.8 Hz) of two anomeric protons allowed the identification of *β*-glucopyranosyl moieties. The HMBC cross peaks (Figure 2) of the anomeric protons *δ* 5.26 (1H, d, *J* = 7.8 Hz, H-1′) and 5.18 (1H, d, *J* = 7.8 Hz, H-1′′) with C-7 (*δ* 141.3) and C-8 (*δ* 136.1), respectively, indicated that two glucopyranosyl groups were linked to C-7 and C-8 of the aglycone, respectively. The methoxy group was located at C-6, based on the HMBC correlation between methoxy group *δ* 3.81 (3H,s) and C-6 (*δ* 149.6). Thus, compound **6** was established as 7,8-bis(*β*-d-glucopyranosyloxy)-6-methoxycoumarin, and named genglycoside F.

According to the previous procedure [22], all isolated compounds were evaluated for cytotoxic activities against Eca-109, MCF-7, and HepG2 cell lines, as well as a normal human umbilical vein endothelial cell line (HUVEC) (Table 3). Etoposide was used as the positive control. All isolated compounds exerted no cytotoxicity against the normal cell line. Compound **12** showed the highest cytotoxicity against Eca-109, MCF-7, and HepG2 cell lines, with IC_50_ values of 20.38, 28.61, 30.27 μM, respectively. Compounds **1**–**10** and **15**–**16** had no cytotoxicity with IC_50_ > 100 μM. Furthermore, 7,8-dihydroxy derivative **12** exhibited significantly higher activity compared to corresponding glycosylation and etherification analogues **1**, **2**, **4–6**, **9**, **11**, **14**, and **15**, indicating that 7,8-dihydroxy were structurally required for the cytotoxicity against Eca-109, MCF-7, and HepG_2_ cells lines. The same effect was found between 7-hydroxy derivative **13** and corresponding analogues **3**, **7**, **10** and **16**. The glycosylation or esterification at 7,8-dihydroxy or 7-dihydroxy drastically reduced the cytotoxic activity of the parent coumarin. With the promising cytotoxicities against three cell lines, compound **12** may be the most valuable lead compound in all tested isolates.

## 3. Experimental Section

### 3.1. General Experimental Procedures

The UV spectra were measured on a Shimadzu UV-1700 spectrometer (Shimadzu Corporation, Kyoto, Japan). The IR spectra were measured on a Nicolet 10 Microscope Spectrometer (Thermo Scientific, San Jose, CA, USA). The 1D and 2D NMR spectra were recorded on Bruker-AC (E)-500 spectrometer (Bruker AM 500, Fällanden, Switzerland) using TMS as an internal standard. The HR-ESI-MS was determined on a Bruker microTOF-Q instrument (Bruker BioSpin, Rheinstetten, Germany). Column chromatography was performed with silica gel (200–300 mesh; Qingdao Marine Chemical Inc., Qingdao, China), sephadex LH-20 (GE Healthcare), ODS (50 µm; YMC Co. LTD., Kyoto, Japan.), AB-8 macroporous resin (Qinshi Science and Technology Ltd., Zhengzhou, China). Thin layer chromatography (TLC) was carried out on silica gel GF254 precoated plates (Qingdao Marine Chemical Inc., Qingdao, China), and spots were visualized under UV light. Preparative HPLC separations were performed on a SEP system (Beijing Sepuruisi scientific Co., Ltd., China) equipped with a variable-wavelength UV detector, using a YMC-Pack ODS-A column (250 × 20 mm, 5 μm). Chemical reagents for isolation were of analytical grade and purchased from Tianjin Siyou Co., Ltd., China. Biological reagents were from Sigma Company. Human heptocellular (HepG2), esophageal (Eca-109), and breast (MCF-7) cancer cell lines were from Institute of Materia Medica, Chinese Academy of Medical Sciences and Peking Union Medical College, China.

### 3.2. Plant Material

The plant materials were collected from Liping, Guizhou province, China, in September 2016, and identified by Cheng-Ming Dong as the aerial parts of *G. vulgaris*, according to the *Chinese Pharmacopoeia 2015*. A voucher specimen (GV 20160901) was deposited at the School of Pharmacy, Henan University of Chinese Medicine.

### 3.3. Extraction and Isolation

The dried aerial parts of *G. vulgaris* were ground into a power (20 kg) and refluxed with 95% EtOH (60 L × 3). The filtrate was concentrated under reduced pressure to yield a dark-brown residue (1.1 kg). The residue was suspended in water (4.4 L) and partitioned with petroleum ether (PE, 4.4 L × 3), EtOAc (4.4 L × 3), and *n*-BuOH (4.4 L × 3), successively.

The EtOAc extract (194 g) was fractionated using silica gel column chromatography (CC, 11 × 70 cm) with a gradient of PE (60–90 °C)–acetone. The fractions were combined into ten main fractions E1–10 based on TLC results. Fraction E8 (3.05 g) was chromatographed over open ODS (2.5 × 45 cm) eluted by methanol–H_2_O (v/v 10:90, 20:80, 30:70, 40:60, 50:50, 60:40, 70:30) to yield sub-fractions E–8–1~E–8–3. Sub-fraction E–8–2 (0.95 g) was further submitted to silica gel CC (1.0 × 20 cm) eluted by CHCl_3_–MeOH (100:10) to give **15** (3.1 mg). Sub-fraction E–8–3 (1.44 g) was further applied to preparative HPLC, eluted with methanol–H_2_O (75: 25) at a flow rate of 7 mL/min to give **13** (7.8 mg, t_R_ 15 min), **14** (5.1 mg, t_R_ 18 min), **16** (3.5 mg, t_R_ 26 min). Fraction E9 (2.75 g) was further chromatographed over open ODS (2 × 40 cm) eluted with a gradient of methanol–H_2_O (v/v 30:70, 60:40, 65:35, 70:30, 80:20, 90:10) to yield sub-fractions E–9–1~E–9–2. Sub-fraction E–9–1 (0.85 g) was purified by preparative HPLC eluted with methanol–H_2_O (70:30) at a flow rate of 7 mL/min to give **12** (82.6 mg, t_R_ 22 min).

The *n*-BuOH extract (64 g) was fractionated by AB-8 CC (5 × 90 cm, 900 g) with a gradient system (EtOH-H_2_O; 0:100, 30:70, 95:5, each 5 L) to give fractions N1 and N2. Fraction N1 (15 g) was separated by silica gel CC (5 × 50 cm, 150 g) with a gradient system of increasing polarity (CH_2_Cl_2_–MeOH; 100:3, 100:5, 100:7, 100:10, 100:20, 100:30, 100:50) to afford sub-fraction N-1-1~N-1-3. Sub-Fraction N-1-1 (1.5 g) was subjected to sephadex LH-20 CC (2 × 50 cm) eluted by methanol to give compounds **4** (4.2 mg) and **10** (3.9 mg). Sub-Fraction N-1-3 (1.2 g) was purified by preparative HPLC eluted with MeOH–H_2_O (40: 60) at 7 mL/min to yield **1** (10.5 mg, t_R_ 12 min), **5** (7.6 mg, t_R_ 16 min), **6** (5.3 mg, t_R_ 18 min), **3** (4.5 mg, t_R_ 21 min), **7** (7.9 mg, t_R_ 25 min). Fraction N2 (6 g) was separated by sephadex LH-20 CC (2.0 × 90 cm), eluted by methanol to yield sub-fraction N-2-1~ N-2-5. Sub-fraction N-2-1 (1.82 g) was purified by silica gel CC (1.5 × 22 cm, 18 g) eluted with CH_2_Cl_2_–MeOH (100:3, 100:5, 100:7, 100:10, 100:30) to compounds **2** (3.8 mg), **8** (4.3 mg), **9** (3.1 mg), **11** (3.5 mg).

### 3.4. Spectroscopic and Physical Data

Genglycoside A (**1**): white, amorphous powder; UV (MeOH) λmax (log *ε* ) 207 (0.73), 291 (0.16), 335 (0.14) nm; IR *ν*_max_ 3384, 2923, 2853, 1702, 1684, 1610, 1509, 1455, 1416, 1271, 1163, 1121 cm^−1^; HR-ESI-MS (positive): *m*/*z* 721.1381 [M + K]^+^ (calcd. for C_30_H_34_O_18_K, 721.1382); NMR data (DMSO-*d_6_*), see Table 1 and Table 2.

Genglycoside B (**2**): white, amorphous powder; UV (MeOH) *λ*max (log *ε* ) 205 (1.08), 295 (0.22), 345 (0.19) nm; IR *ν*_max_ 3359, 2920, 2851, 1710, 1602, 1505, 1418, 1286, 1217, 1124, 1073 cm^−1^; HR-ESI-MS (positive): *m*/*z* 543.1097 [M + Na]^+^ (calcd. for C_24_H_24_O_13_Na, 543.1115); NMR data (DMSO-*d_6_*), see Table 1 and Table 2.

Genglycoside C (**3**): white, amorphous powder; UV (MeOH) *λ*max (log *ε* ) 204 (1.34), 290 (0.26), 340 (0.27) nm; IR *ν*_max_ 3333, 2922, 2853, 1700, 1612, 1564, 1512, 1422, 1394, 1281, 1248, 1199, 1076 cm^−1^; HR-ESI-MS (positive): *m*/*z* 539.1396 [M + Na]^+^ (calcd. for C_22_H_28_O_14_Na, 539.1377); NMR data (DMSO-*d_6_*), see Table 1 and Table 2.

Genglycoside D (**4**): white, amorphous powder; UV (MeOH) *λ*max (log *ε* ) 208 (1.25), 292 (0.17), 343 (0.20) nm; IR_νmax_ 3377, 2927, 2854, 1709, 1603, 1577, 1501, 1456, 1416, 1336, 1221, 1187, 1127, 1067 cm^−1^; HR-ESI-MS (positive): *m*/*z* 719.1794 [M + Na]^+^ (calcd. for C_31_H_36_O_18_Na, 719.1800); NMR data (CD_3_OD), see Table 1 and Table 2.

Genglycoside E (**5**): white, amorphous powder; UV (MeOH) *λ*max (log *ε* ) 206 (1.09), 292 (0.30), 339 (0.20) nm; IR_νmax_ 3378, 2922, 2854, 1706, 1607, 1566, 1509, 1439, 1412, 1348, 1296, 1197, 1163 cm^−1^; HR-ESI-MS (positive): *m*/*z* 555.1320 [M + Na]^+^ (calcd. for C_22_H_28_O_15_Na, 555.1326); NMR data (DMSO-*d_6_*), see Table 1 and Table 2.

Genglycoside F (**6**): white, amorphous powder; UV (MeOH) *λ*max (log *ε* ) 207 (1.20), 292 (0.31), 338 (0.21) nm; IR *ν*_max_ 3327, 2924, 2853, 1682, 1608, 1569, 1506, 1487, 1439, 1414, 1294, 1207, 1137, 1072 cm^−1^; HR-ESI-MS (positive): *m*/*z* 555.1320 [M + Na]^+^ (calcd. for C_22_H_28_O_15_Na, 555.1326); NMR data (DMSO-*d_6_*), see Table 1 and Table 2.

### 3.5. Acid Hydrolysis and Sugar Analysis

According to the literature [23], the absolute configurations of the monosaccharide moieties were determined. Compounds **1**–**6** (2 mg) were hydrolyzed with 2 N HCl (5 mL) for 2 h at 90 °C. The HCl was removed by evaporation, and then extracted by EtOAc (7 mL × 3). The aqueous layer was evaporated to dryness under N_2_ to give a residue. The residue was dissolved in 0.5 mL anhydrous pyridine containing 2 mg l-cysteine methyl ester hydrochloride. The mixture was kept at 60 °C for 1 h, and 20 μL of isothiocyanate was added, followed by heating at 60 °C for another 1 h. Then, the reactant was analyzed by an HPLC system (column: YMC-Triart C18 column (250 × 4.6 mm, 5 μm); eluent: CH_3_CN/0.1%H_3_PO4; detection wavelength: 250 nm, injection volume: 10 μL; flow rate: 1.2 mL/min). The derivatives of d-glucose and l-rhamnose in compounds **1**−**6** were identified by comparison to the retention times of authentic samples (t_R_: d-glucose, 17.4 min; l-rhamnose, 26.9 min).

## 4. Conclusions

With fewer adverse effects, natural products have played an important role in new drug discovery. Extensive research has been focused on natural products with significant cytotoxic activities, such as alkaloids, terpenoids [24], flavonoids [25], and lignans [26]. In contrast, the cytotoxic activities of simple coumarins are rarely reported. Our research revealed that simple coumarins were the major active constituents of *G. vulgaris*. However, until now, the chemical investigations of *G. vulgaris* were inadequate, with only two studies [1,27] indicating that it contained seven alkaloids, eight flavonoids, three phenylpropanoids, two steroids, and one triterpene. The phytochemical investigation of *G. vulgaris* resulted in the isolation of sixteen simple coumarins, including six new glucoside derivatives (**1**–**6**). Among the sixteen isolated coumarins, only compounds **11**–**14** showed certain cytotoxic activities against Eca-109, MCF-7, and HepG2 cell lines. On the basis of the preliminary structure–activity relationship (SAR) studies, 7,8-dihydroxy and 7-hydroxy play a very important role in maintaining cytotoxicity for simple coumarin. The glycosylation and etherification of the 7,8-dihydroxy and 7-hydroxy strongly reduced the cytotoxic activity. This study not only enriches the chemical diversity of coumarin glycosides in *Gendarussa* plants, but also broadens the application field of *G. vulgaris*.

## Figures and Tables

**Table 1:** ^1^H NMR spectroscopic data (500 MHz) of **1**–**6**.

No.	1 ^a^	2 ^a^	3 ^a^	4 ^b^	5 ^a^	6 ^a^
3	6.13 d (9.5)	6.08 d (9.5)	6.33 d (9.6)	5.97 d (9.3)	6.22 d (9.5)	6.39 d (9.5)
4	7.80 d (9.5)	7.79 d (9.5)	7.96 d (9.6)	7.56 d (9.3)	7.88 d (9.5)	7.94 d (9.5)
5	6.93 s	6.92 s	7.30 s	6.65 s	7.02 s	7.14 s
8			7.17 s			
6-OCH_3_	3.77 s	3.76 s	3.81 s	3.80 s	3.81 s	3.81 s
1′	5.06 d (5.3)	4.94 d (7.7)	5.21d (7.4)	5.11 d (7.8)	4.97 d (7.8)	5.26 d (7.8)
2′	3.47 m	3.44 m	3.50 m	3.59 m	3.37 m	3.38 m
3′	3.31 m	3.31 m	3.50 m	3.60 m	2.97 m	3.20 m
4′	3.23 m	3.23 m	3.23 m	4.30 m	3.24 m	3.10 m
5′	3.49 m	3.47 m	3.47 m	3.51 m	2.89 m	3.09 m
6′	4.43 dd (11.8, 2.0) 4.19 dd (11.8, 7.5)	4.46 dd (11.8, 1.9) 4.14 dd (11.8, 7.5)	3.61 m 3.48 m	4.60 m 4.41 m	3.87 m 3.60 m	3.58 m 3.41 m
1′′			4.35 d (7.8)		4.06 d (7.8)	5.18 d (7.8)
2′′	7.28 d (1.9)	7.29 d (1.9)	3.10 m	7.03 s	2.84 m	3.38 m
3′′			3.21 m		3.24 m	3.20 m
4′′			3.02 m		2.99 m	3.10 m
5′′	7.05 d (8.5)	6.79 d (8.2)	3.23 m		3.32 m	3.12 m
6′′	7.18 dd (8.5, 1.9)	7.22 dd (8.2, 1.9)	3.61 m 3.43 m	7.03 s	3.38 m 3.57 m	3.58m 3.40 m
3′′-OCH_3_	3.74 s	3.75 s		3.79 s		
5′′-OCH_3_				3.79 s		
1′′′	5.12 d (5.3)			5.38 br.s		
2′′′	3.25 m			3.89 m		
3′′′	3.32 m			3.48 m		
4′′′	3.22 m			3.40 m		
5′′′	3.47 m			4.15 m		
6′′′	3.71 m 3.48 m			1.25 d (6.2)		

*^a^*^1^H NMR data (*δ*) were measured in DMSO-*d*_6_; *^b^*^1^H NMR data (*δ*) were measured in CD_3_OD.

**Table 2:** ^13^C NMR Spectroscopic Data (100 MHz) of **1**–**6**.

No.	1^a^	2 ^a^	3 ^a^	4 ^b^	5 ^a^	6 ^a^	No.	1 ^a^	2 ^a^	3 ^a^	4 ^b^	5 ^a^	6 ^a^
2	160.1 s	160.2 s	160.4 s	163.4 s	160.2 s	159.9 s	1′′	122.8 s	123.4 s	103.9 d	126.9 s	103.0 s	102.5 d
3	111.9 d	115.0 d	113.4 d	112.4 d	111.2 d	114.9 d	2′′	112.4 d	112.4 d	73.8 d	107.6 d	73.5 d	74.0 d
4	144.6 d	144.5 d	144.1 d	146.0 d	144.8 d	144.2 d	3′′	148.4 s	147.2 s	75.9 d	154.3 s	76.2 d	76.4 d
5	104.8 d	103.8 d	110.0 d	105.3 d	105.0 d	105.9 d	4′′	150.5 s	151.4 s	70.1 d	139.7 s	69.8 d	69.9 s
6	145.3 s	145.8 s	146.0 s	147.2 s	145.3 s	149.6 s	5′′	114.0 d	115.0 d	76.9 d	154.3 s	76.6 d	77.5 d
7	148.4 s	147.2 s	148.9 s	146.0 s	143.7 s	141.3 s	6′′	122.6 d	120.5 d	60.4 t	107.6 d	60.8 d	60.7 t
8	131.3 s	131.5 s	103.1d	132.2 s	131.3 s	136.1 s	7′′	165.0 s	165.3 s		167.0 s		
9	142.8 s	143.2 s	148.9 s	144.6 s	142.7 s	142.4 s	3′′-OCH_3_	55.5 q	55.5 q		56.5 q		
10	110.0 s	112.4 s	112.4 s	110.2 s	110.1 s	114.4 s	5′′-OCH_3_				56.5 q		
6-OCH_3_	56.1 q	56.0 q	56.1 q	56.8 q	56.1 q	56.6 q	1′′′	99.6 d	99.6 d		103.4 d		
1′	103.2 d	104.8 d	99.0 d	104.4 d	103.6 d	102.6 d	2′′′	73.1 d	73.1 d		72.3 d		
2′	73.7 d	73.8 d	71.8 d	75.3 d	73.8 d	74.0 d	3′′′	76.8 d	76.8 d		73.6 d		
3′	76.2 d	76.3 d	87.4 d	77.9 d	76.4 d	76.4 d	4′′′	69.7 d	69.7 d		72.2 d		
4′	70.4 d	70.2 d	67.9 d	71.4 d	69.5 d	69.9 d	5′′′	77.3 d	77.3 d		72.0 d		
5′	74.2 d	74.3 d	76.5 d	75.7 d	76.5 d	77.6 d	6′′′	60.8 t	60.8 t		18.0 q		
6′	64.0 t	63.7 t	61.1 t	65.2 t	67.7 t	60.7 t							

*^a^*^1^H NMR data (*δ*) were measured in DMSO-*d*_6_; *^b^*^1^H NMR data (*δ*) were measured in CD_3_OD.

**Table 3:** Cytotoxicities of compounds **11**–**14** against Eca-109, MCF-7, and HepG2 cell lines (IC_50_, μM).

Compound	Eca-109	MCF-7	HepG2	HUVEC
**11**	21.04 ± 1.85	35.29 ± 2.61	43.72 ± 3.97	>100
**12**	20.38 ± 1.94	28.61 ± 1.37	30.27 ± 1.18	>100
**13**	45.72 ± 3.55	61.59 ± 5.70	53.74 ± 4.09	>100
**14**	41.09 ± 3.78	59.59 ± 5.24	>100	>100
etoposide	20.48 ± 1.82	5.82 ± 0.49	1.15 ± 0.09	41. 65 ± 0.32

## Supplementary Materials

The following are available online. Figures S1–S24: NMR spectra of compounds **1**–**6**.
