# Supplementary material for: Six New Coumarin Glycosides from the Aerial Parts of Gendarussa vulgaris
Source: Molecules. 2019 Apr 12;24(8):1456. doi: 10.3390/molecules24081456 (PMC6514664; doi:10.3390/molecules24081456)
Supplement: Supplementary file 1 [file molecules-24-01456-s001.pdf]

Supporting Information

Six New Coumarin Glycosides from the Aerial Parts of *Gendarussa vulgaris*

Yanjun Sun [1,2,*], Meiling Gao [1,2], Haojie Chen [1,2], Ruijie Han [1,2], Hui Chen [1,2], Kun Du [1,2], Yanli Zhang [1,2], Meng Li [1,2], Yingying Si [1,2] and Weisheng Feng [1,2,*]

[1] Collaborative Innovation Center for Respiratory Disease Diagnosis and Treatment & Chinese Medicine Development of Henan Province, Henan University of Chinese Medicine, Zhengzhou 450046, Henan, China; gaoxiaomei6266@126.com (M.G.); CHj3928@126.com (H.C.); 18638221936@126.com (R.H.); chenhuiyxy@hactcm.edu.cn (H.C.); qqninenine@hotmail.com (K.D.); zyl2013hnzy@163.com (Y.Z.); limeng31716@163.com (M.L.); yingying8690@163.com (Y.S.)

[2] School of Pharmacy, Henan University of Chinese Medicine, Zhengzhou 450046, Henan, China

* Correspondence: sunyanjun2011@hactcm.edu.cn (Y.S.); fwsh@hactcm.edu.cn (W.F.); Tel.: +86-371-6596-2746 (Y.S. & W.F.).

Content

The 1D and 2D NMR spectra of compound **1**...3
The 1D and 2D NMR spectra of compound **2**...5
The 1D and 2D NMR spectra of compound **3**...7
The 1D and 2D NMR spectra of compound **4**.. 9
The 1D and 2D NMR spectra of compound **5**...11
The 1D and 2D NMR spectra of compound **6**...13

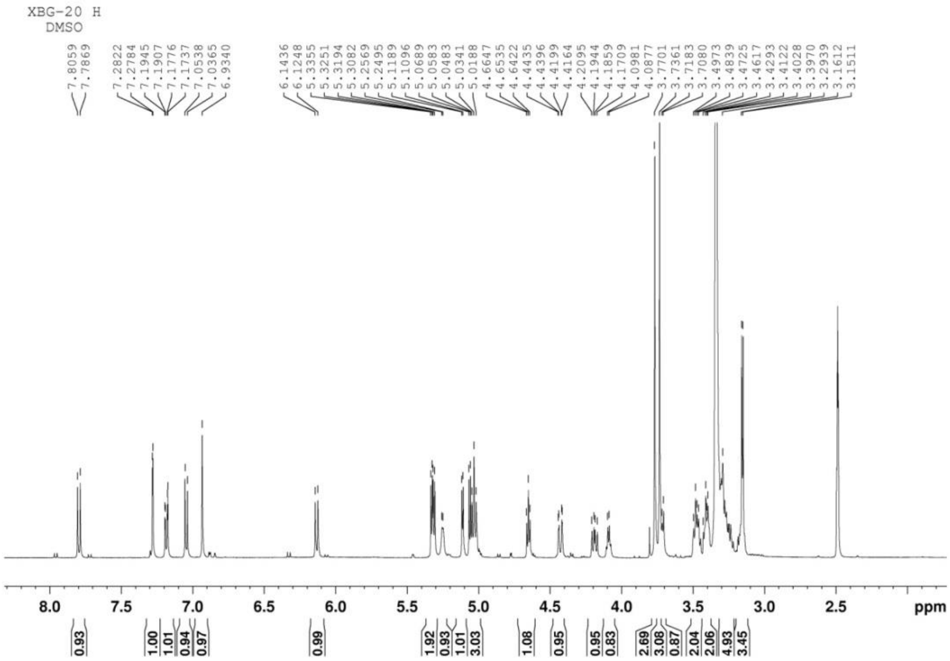

Figure S1. ^{1}H-NMR (500 MHz, DMSO-d_6) spectrum of compound **1**.

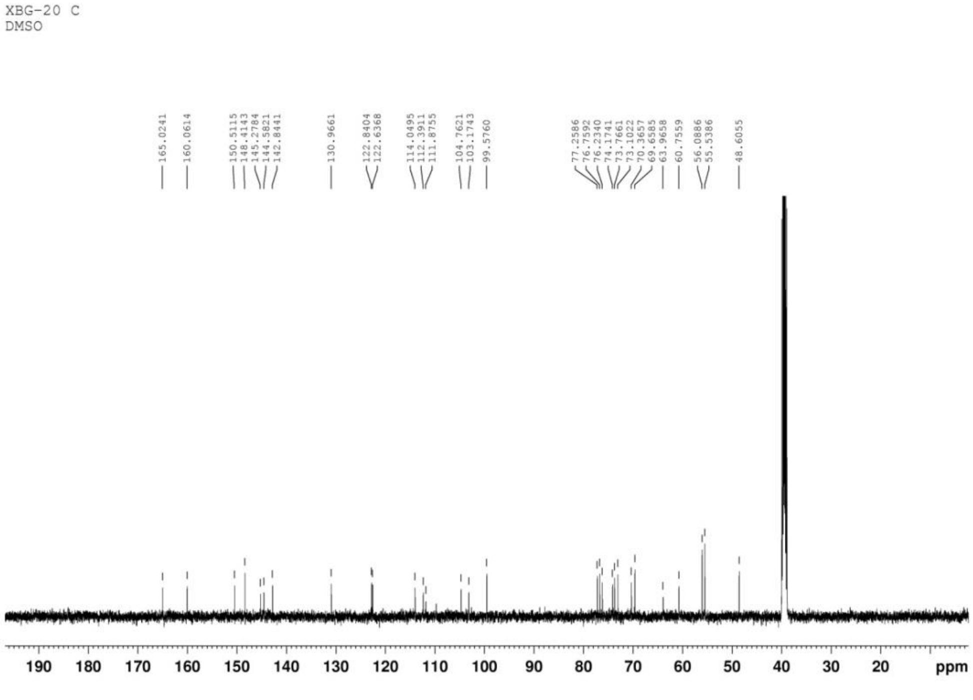

Figure S2. ^{13}C-NMR (125 MHz, DMSO-d_6) spectrum of compound **1**.

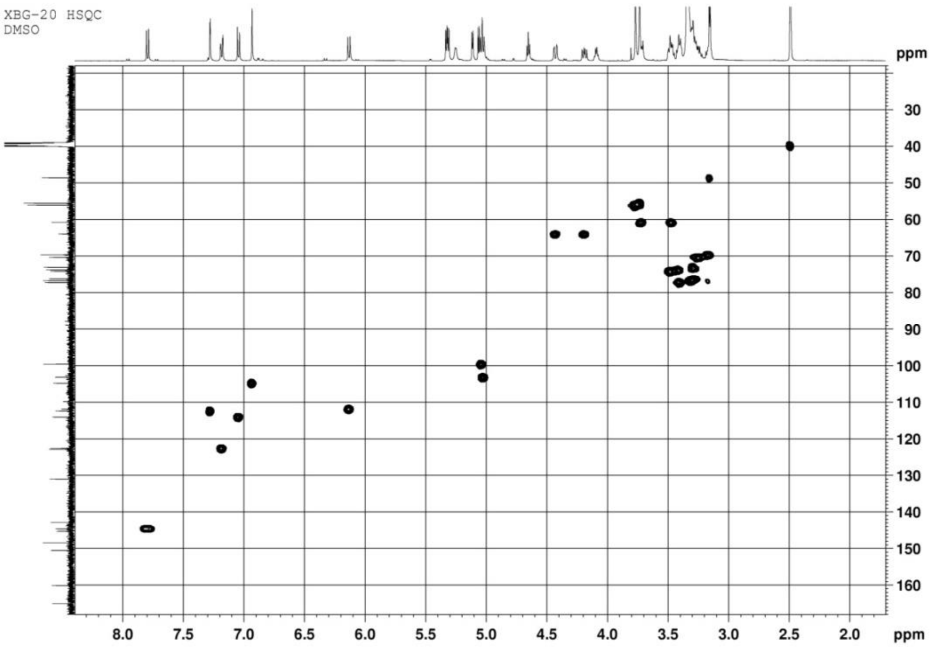

Figure S3. HSQC spectrum of compound **1**.

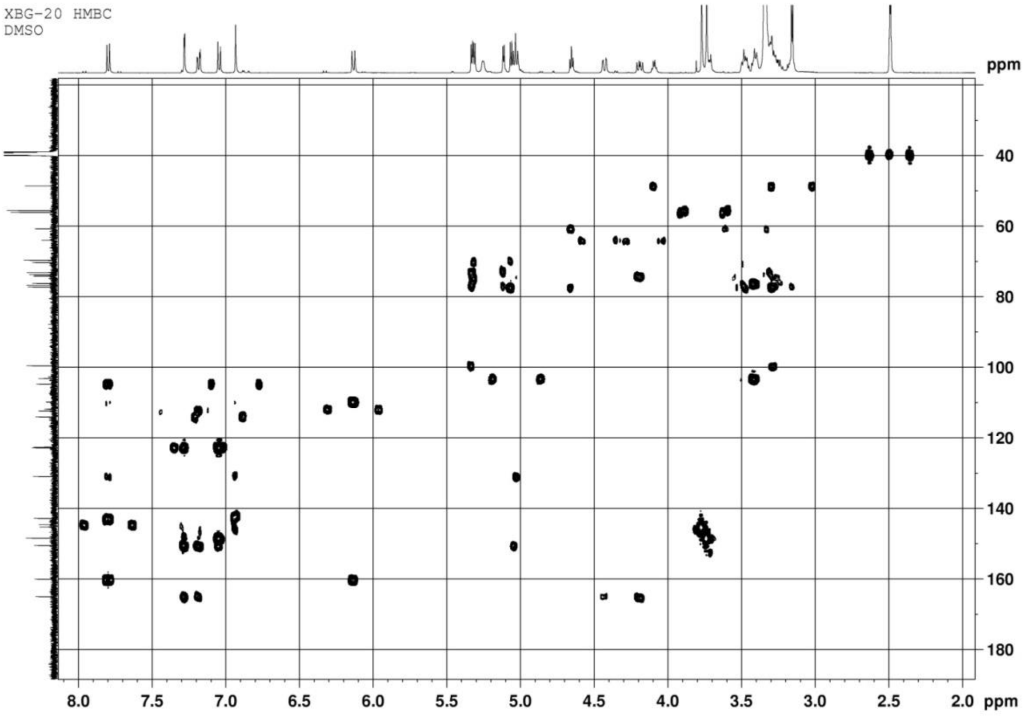

Figure S4. HMBC spectrum of compound **1**.

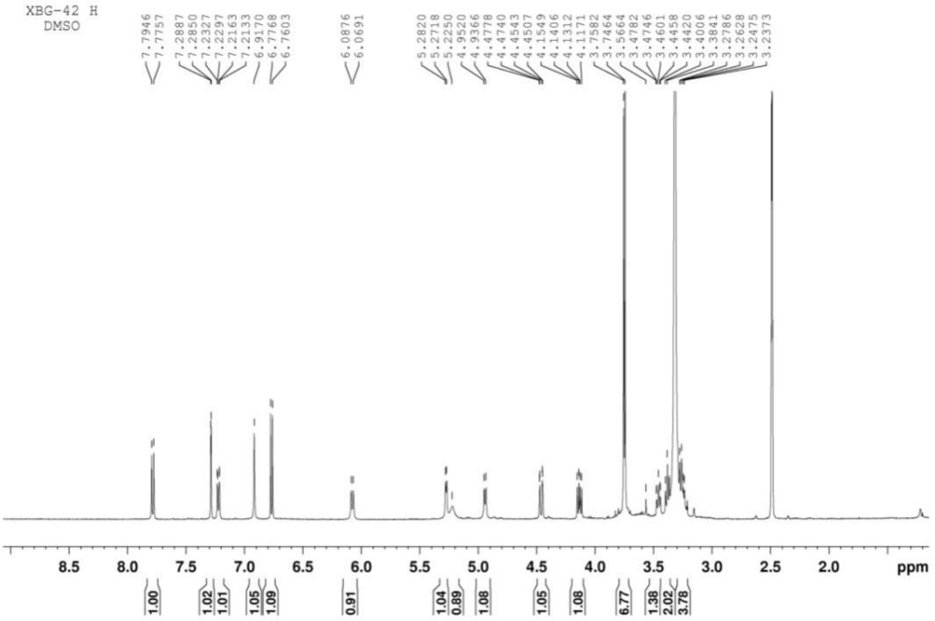

Figure S5. ^{1}H-NMR (500 MHz, DMSO-d_6) spectrum of compound **2**.

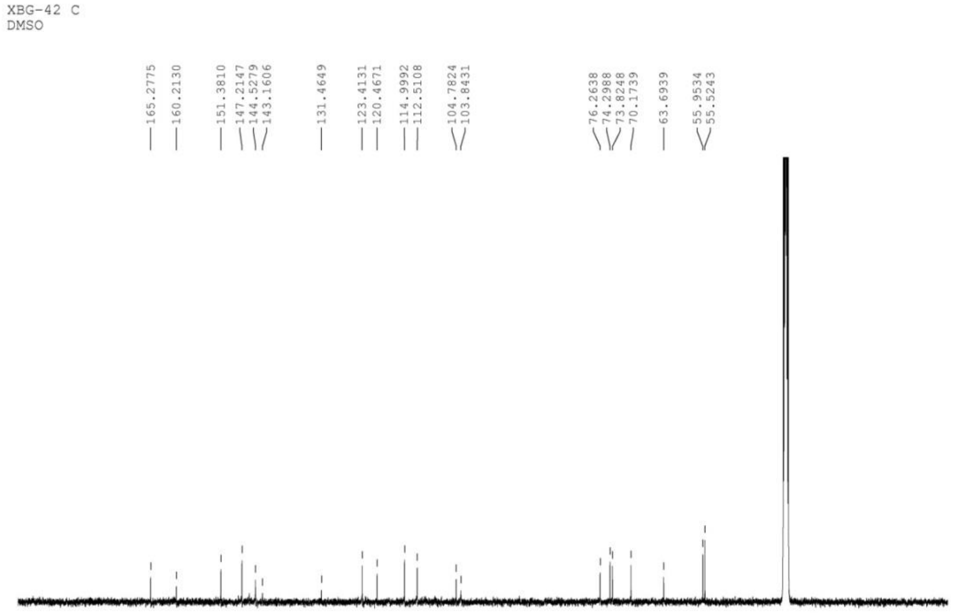

Figure S6. ^{13}C-NMR (125 MHz, DMSO-d_6) spectrum of compound **2**.

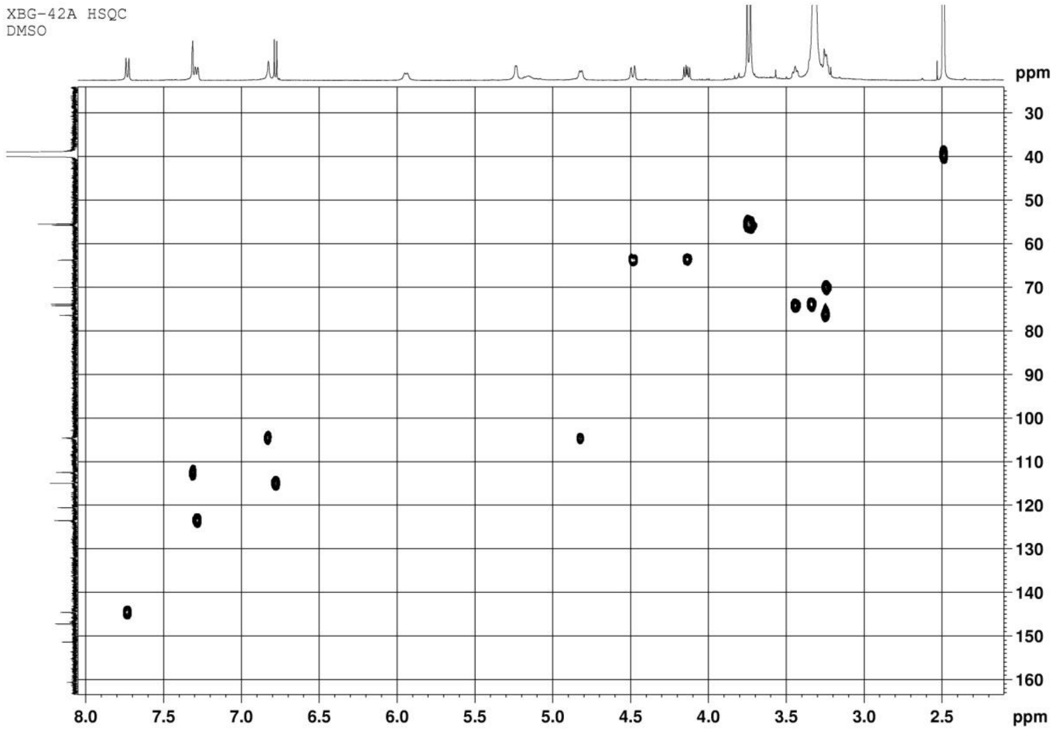

Figure S7. HSQC spectrum of compound **2**.

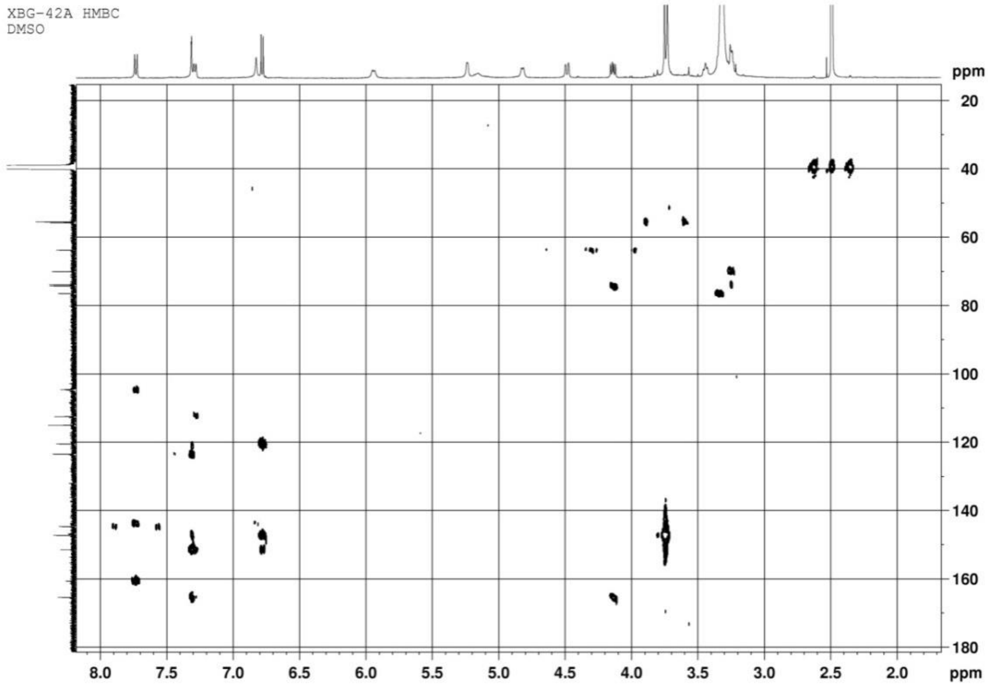

Figure S8. HMBC spectrum of compound **2**.

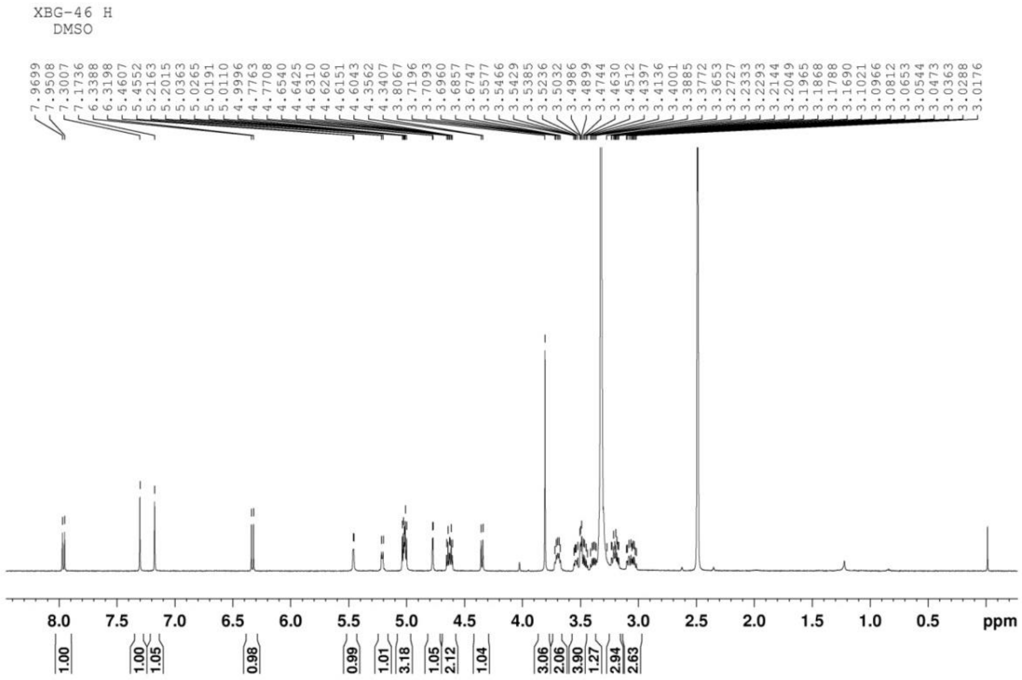

Figure S9. ^{1}H-NMR (500 MHz, DMSO-d_6) spectrum of compound **3**.

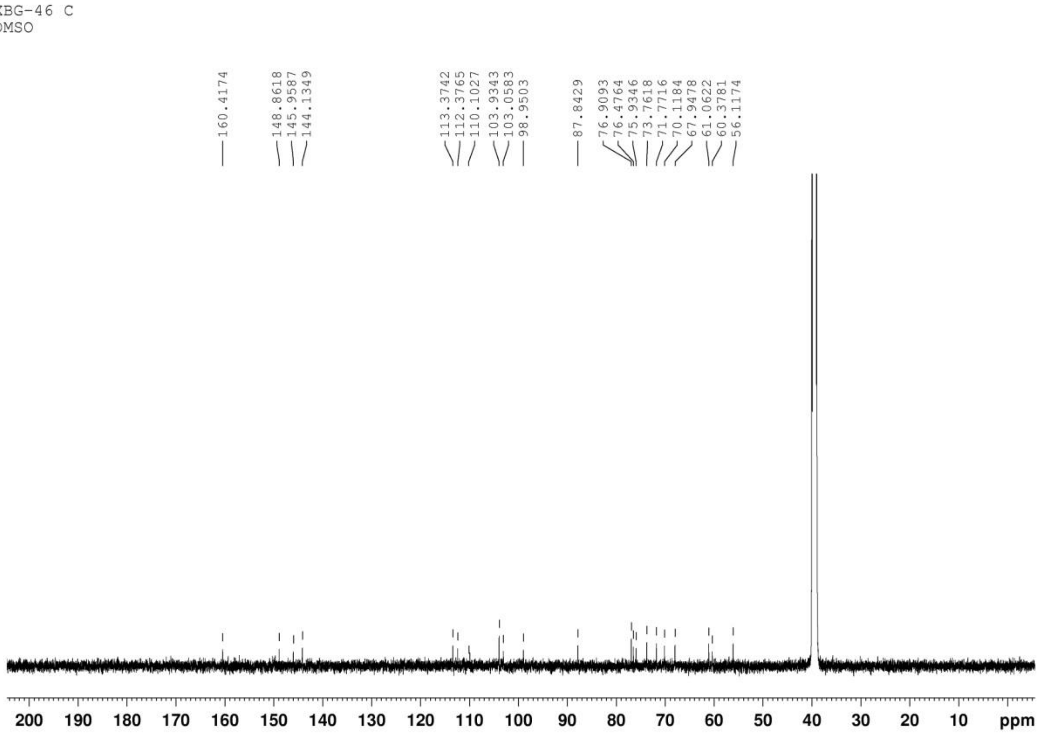

Figure S10. ^{13}C-NMR (125 MHz, DMSO-d_6) spectrum of compound **3**.

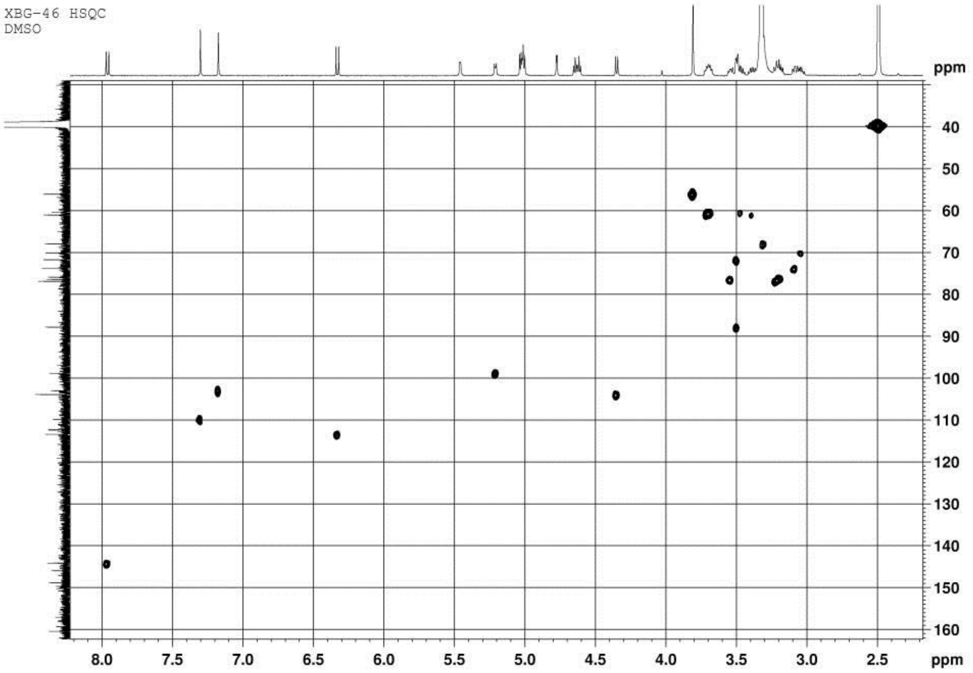

Figure S11. HSQC spectrum of compound **3**.

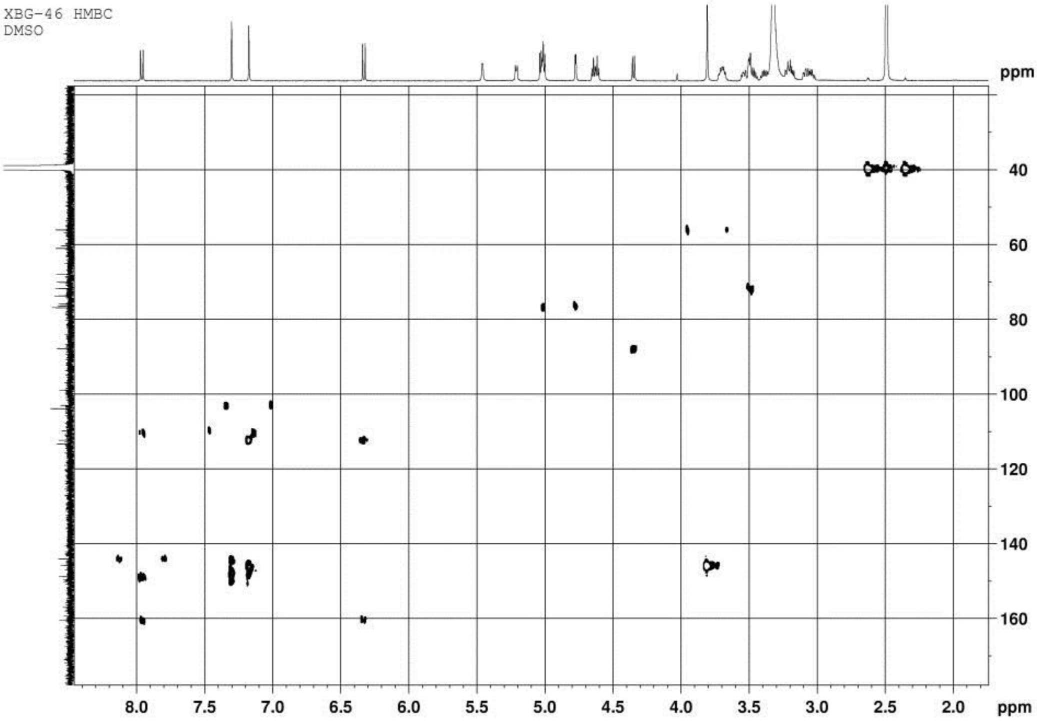

Figure S12. HMBC spectrum of compound **3**.

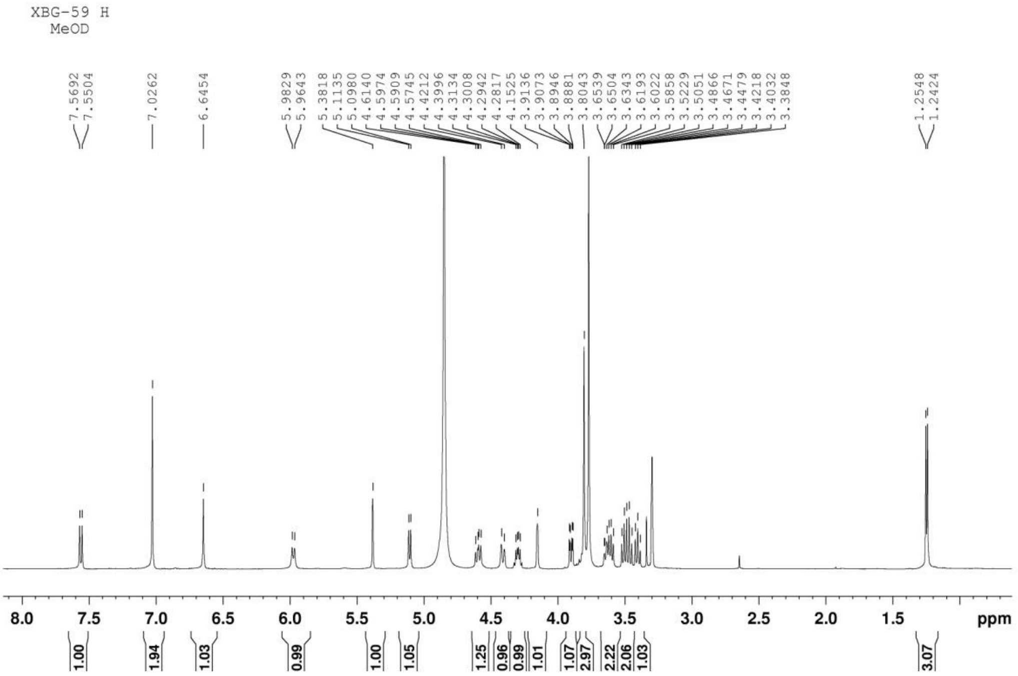

Figure S13. ^{1}H-NMR (500 MHz, CD_3OD) spectrum of compound **4**.

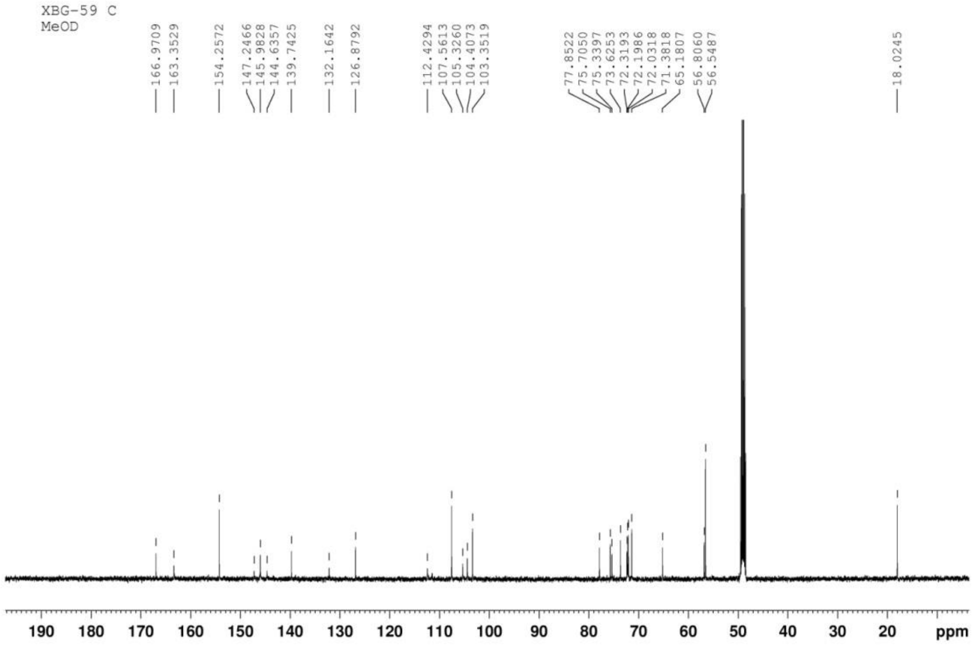

Figure S14. ^{13}C-NMR (125 MHz, CD_3OD) spectrum of compound **4**.

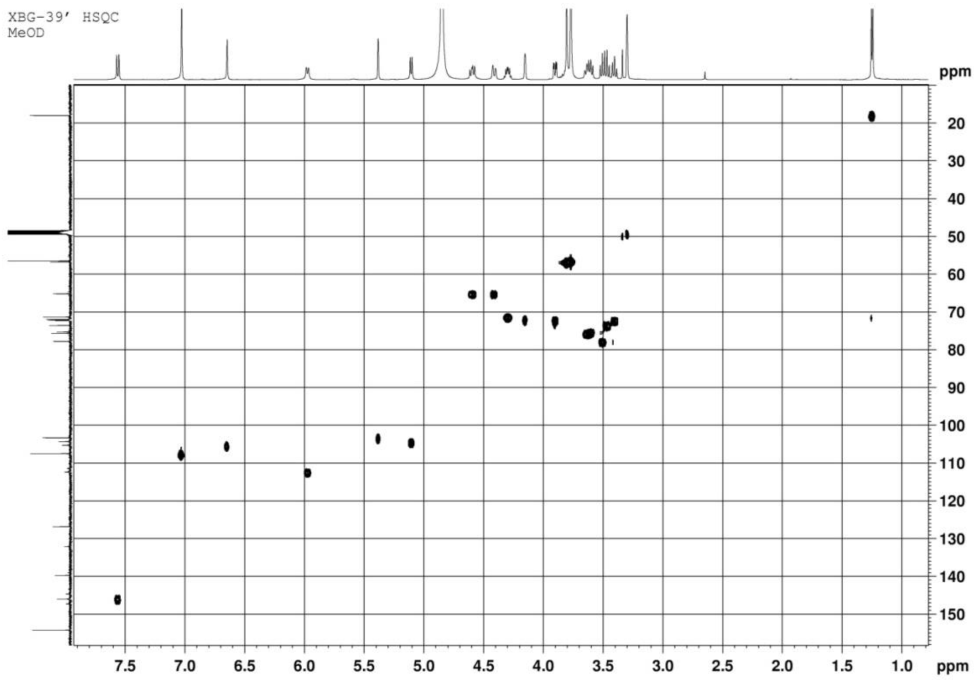

Figure S15. HSQC spectrum of compound **4**.

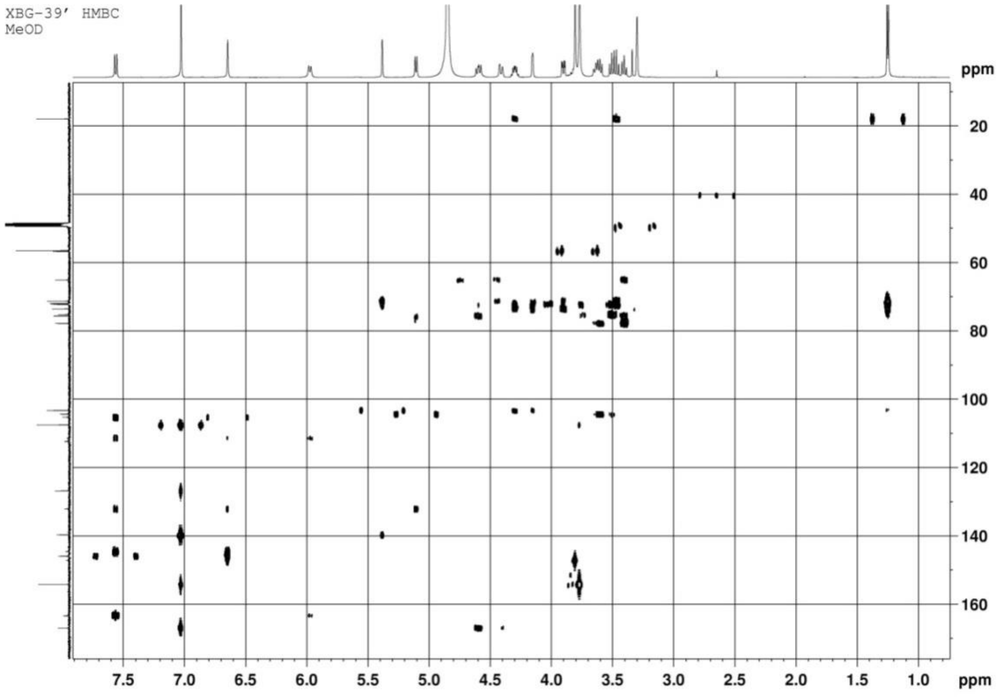

Figure S16. HMBC spectrum of compound **4**.

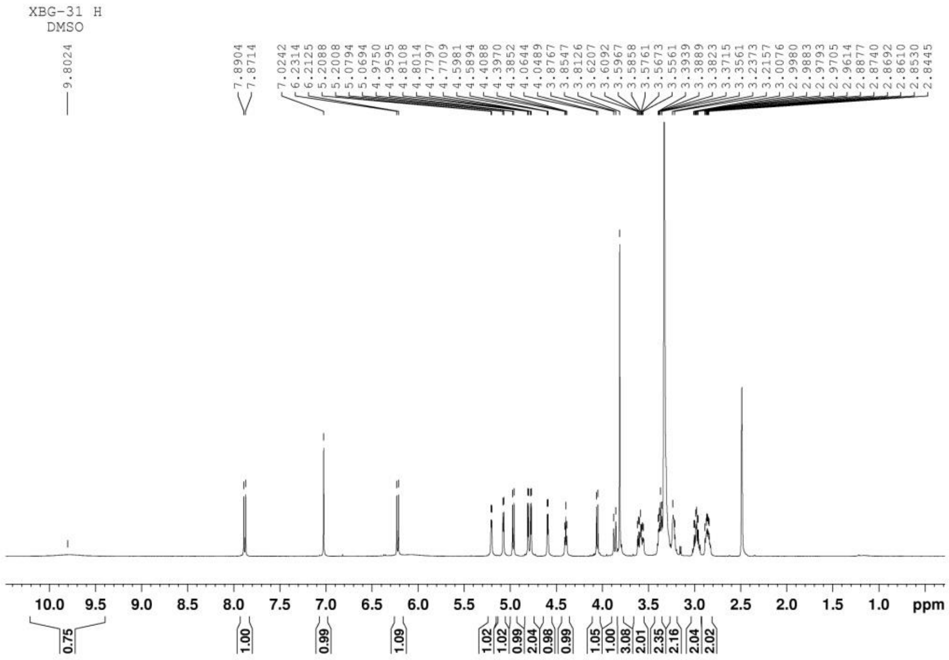

Figure S17. ^{1}H-NMR (500 MHz, DMSO-d_6) spectrum of compound **5**.

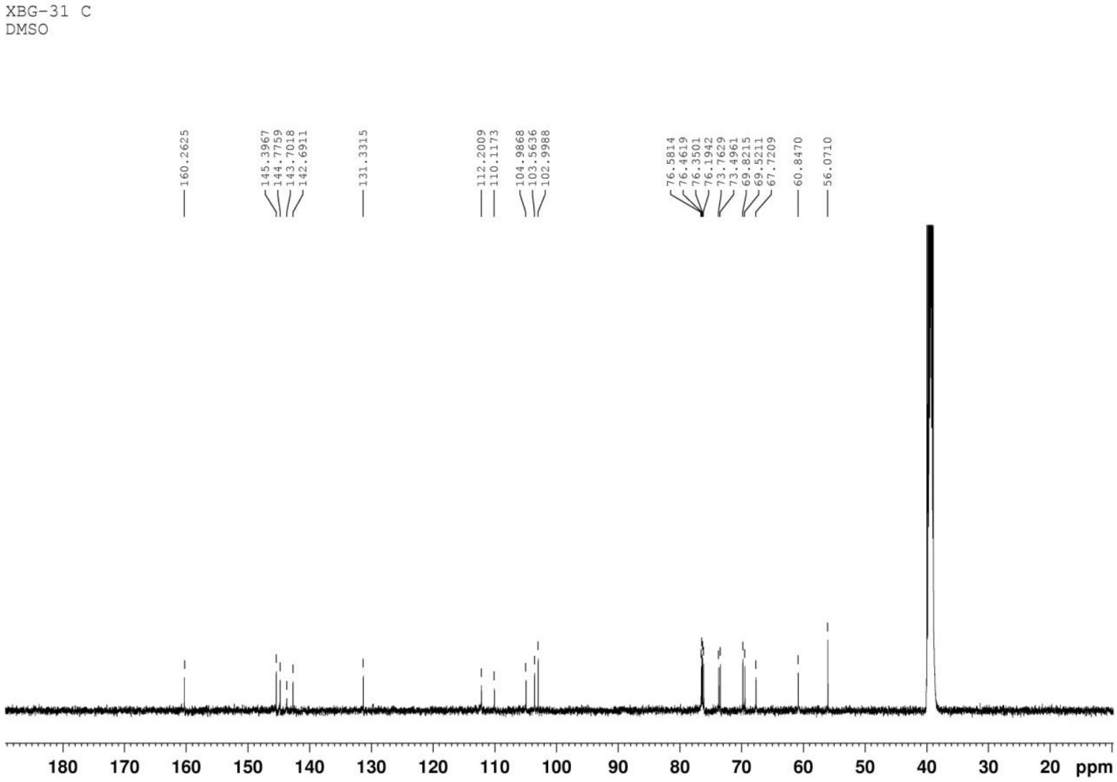

Figure S18. ^{13}C-NMR (125 MHz, DMSO-d_6) spectrum of compound **5**.

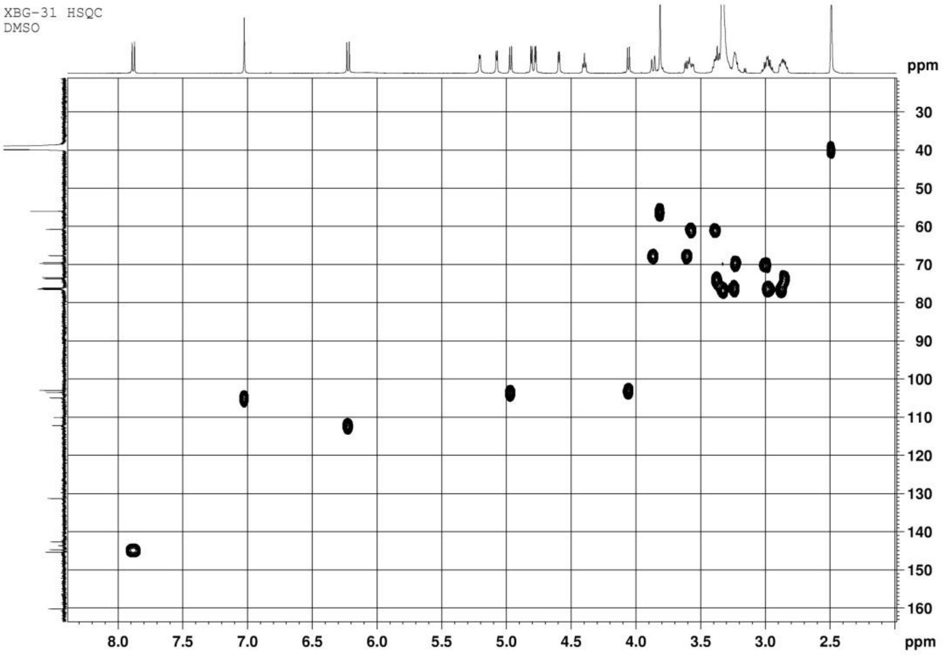

Figure S19. HSQC spectrum of compound **5**.

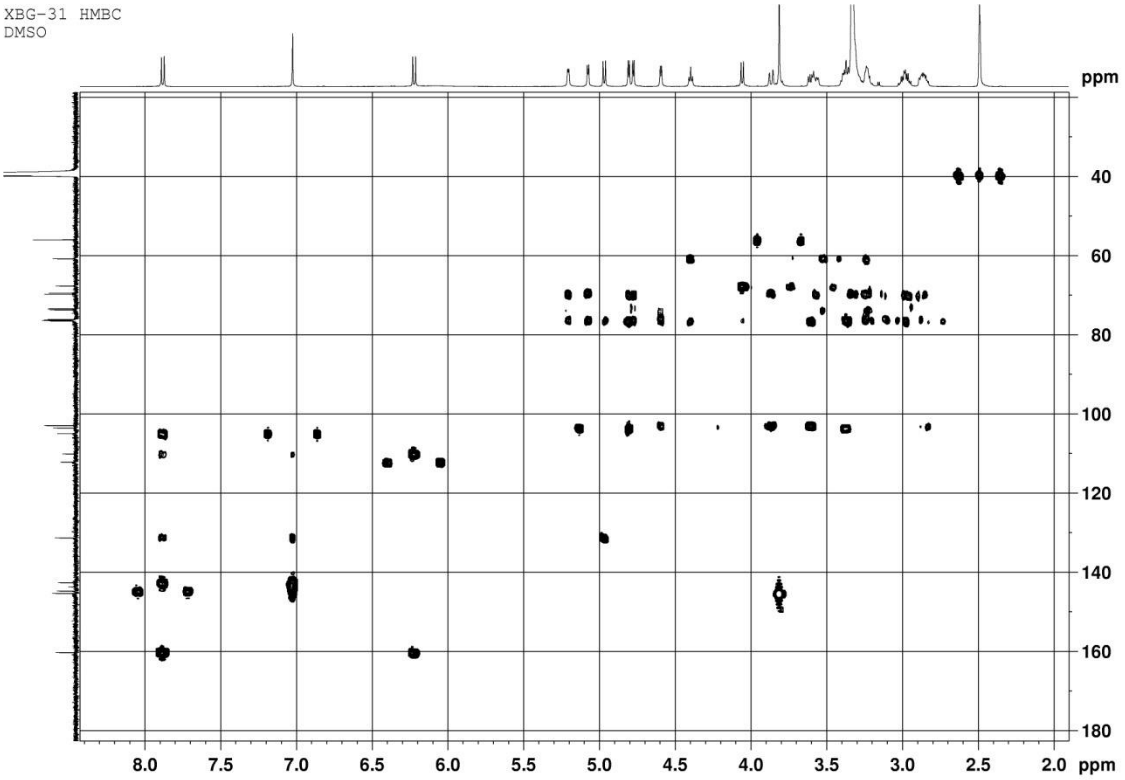

Figure S20. HMBC spectrum of compound **5**.

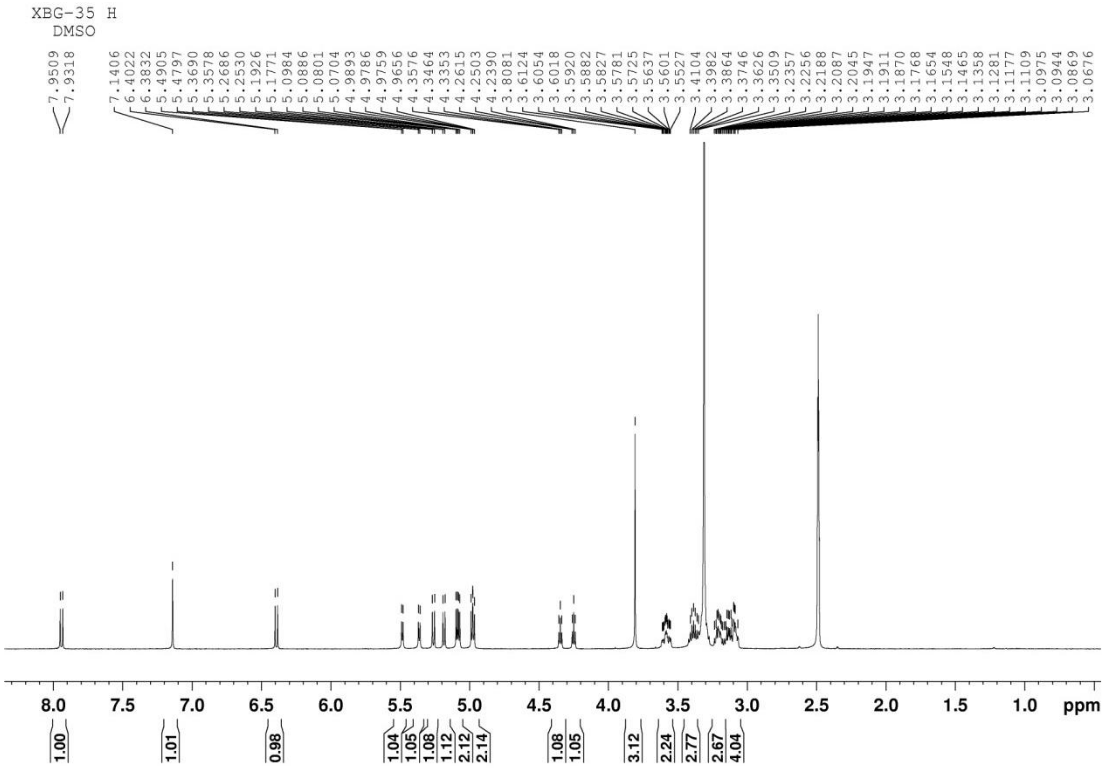

Figure S21. ^{1}H-NMR (500 MHz, DMSO-d_6) spectrum of compound **6**.

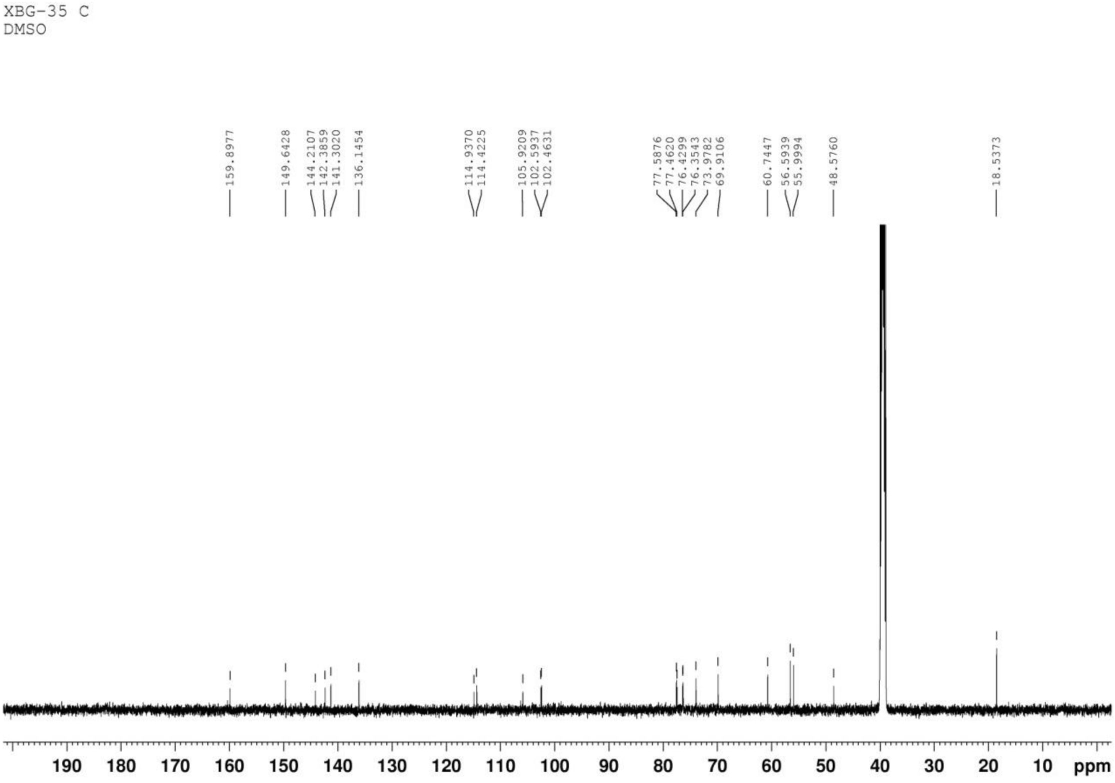

Figure S22. ^{13}C-NMR (125 MHz, DMSO-d_6) spectrum of compound **6**.

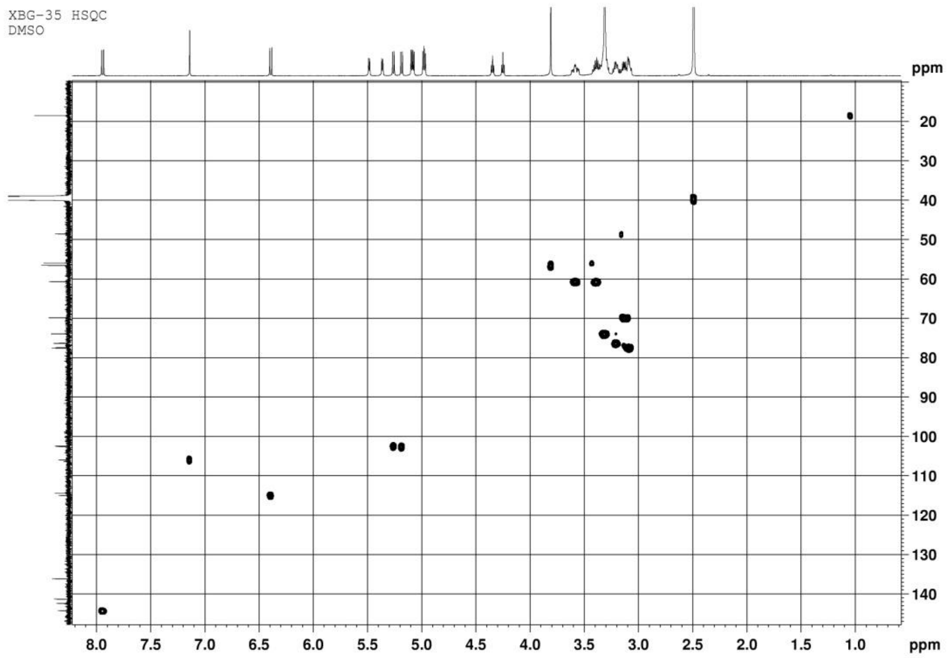

Figure S23. HSQC spectrum of compound **6**.

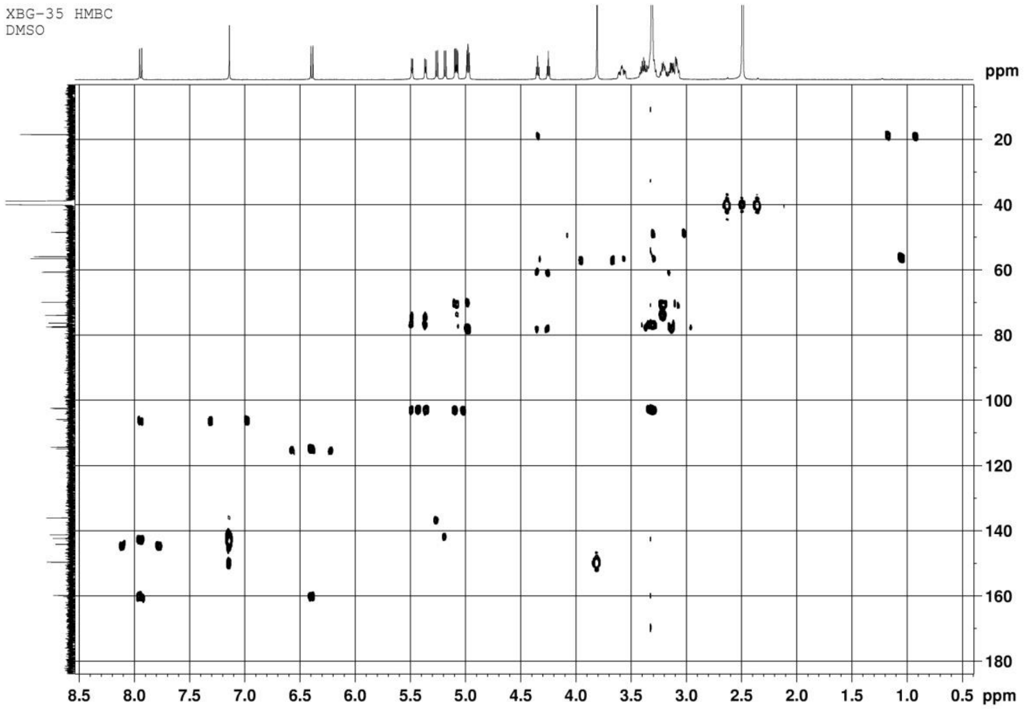

Figure S24. HMBC spectrum of compound **6**.
